# The role of gallic acid in liver disease: a review of its phytochemistry, pharmacology, and safety

**DOI:** 10.3389/fphar.2025.1595508

**Published:** 2025-07-25

**Authors:** Peiyu Li, Yifan Song, Linlin Lv, Wenshuo Zhang, Aixi Jia, Deshi Dong, Xiaohan Zhai

**Affiliations:** Department of Pharmacy, The First Affiliated Hospital of Dalian Medical University, Dalian, China

**Keywords:** gallic acid (GA), hepatoprotective effects, liver diseases, mechanism, signaling pathways

## Abstract

**Background:**

The development of liver diseases adversely affects global health, emerging as a prominent cause of mortality globally and imposing a significant economic strain on society. Gallic acid (GA) is the natural polyphenol that is present in a variety of plants, fruits, tea, traditional Chinese medicine and so on.

**Purpose:**

This review was aimed to analyze the available literature on GA with a focus on its mechanism of action.

**Methods:**

Several literature databases were searched, including PubMed, Web of Science, Google Scholar, and Scopus to find relevant research on GA and liver disease over the last decade.

**Results:**

Our finding indicate that GA can effectively reduce non-alcoholic liver injury, alcoholic liver disease, hepatic fibrosis, drug-induced liver injury, and liver cancer. GA displays remarkable antioxidant effects by activating nuclear factor erythroid 2-related factor (Nrf2) and the expression of antioxidant genes. Moreover, the anti-inflammatory mechanism is mainly related to the nuclear factor kappa B (NF-κB) signaling pathway and down-regulating some inflammation-related factors such as interleukin 1 (IL-1), interleukin 6 (IL-6), transforming growth factor-beta (TGF-β) and tumor necrosis factor alpha (TNF-α). GA mitigates non-alcoholic fatty liver disease (NAFLD) and alcoholic liver disease (ALD) through the reduction of lipid accumulation, achieved by modulating the AMP-activated protein kinase (AMPK) signaling pathway. In the context of liver cancer, GA additionally modulates the wnt/β-catenin and JAK/STAT3 signaling pathways, as well as their downstream molecular components.

**Conclusion:**

In this review, different studies indicate that GA have an excellent protective effect against various liver diseases associated with various signaling pathways.

## Graphical Abstract

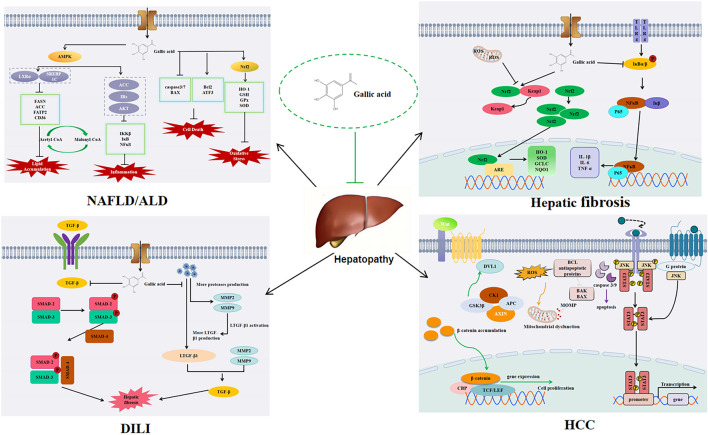

## Introduction

In the human body, the liver performs several essential functions related to substance metabolism, excretion, detoxification, and immunity. Additionally, owing to its diverse roles, the liver is highly prone to damage. Many factors can contribute to liver injury, including drugs (Corsini and Bortolini, 2013), chemicals (Ouyang et al., 2023), alcohol (Chao et al., 2014) and viruses (Li et al., 2022a), as well as bile stasis, autoimmune conditions, genetics, metabolic disorders, and others (Xu et al., 2021a). During the past few decades, liver disease has emerged as one of the primary global causes of disease and mortality, causing over 2 million deaths each year (Rahimifard et al., 2020). The financial burden of treating liver diseases is substantial, with costs varying widely depending on the type and severity of the condition. For instance, fatty liver treatment may cost around $2,000, while viral hepatitis can require approximately $5,000 for treatment and $2,000 annually for medication. More severe cases, such as liver cancer or decompensated cirrhosis, can incur costs ranging from $50,000 to $80,000. In 2016, the United States spent a staggering $32.5 billion on liver disease-related issues. Not only does this impose a heavy burden on the economy, but it also poses a severe threat to people’s health (Devarbhavi et al., 2023; Xiao et al., 2019). Therefore, preserving liver health is essential for overall wellbeing and complication prevention. Scientists are actively developing more effective drugs for liver injury treatment.

Dietary polyphenols have garnered widespread attention due to their role in cancer, cardiovascular diseases, and neurodegenerative disorders (Tsao, 2010). Polyphenols are a type of plant secondary metabolite, widely distributed in various tissues of plants, and possess robust antioxidant properties. Research has shown that polyphenols offer numerous benefits for the liver and its associated complications. In addition to regulating oxidative stress, improving lipid metabolism, enhancing insulin sensitivity, and exhibiting anti-inflammatory effects, and anti-tumor resistance, they also aid in weight management and the management of chronic illnesses (Al-Dashti et al., 2018; Li et al., 2014). There is a wide variety of polyphenolic compounds, with approximately over 8,000 different types, including common compounds such as apigenin, quercetin, curcumin, anthocyanins, gallic acid, and flavonoids (Abenavoli et al., 2021; Jantan et al., 2021; Kawabata et al., 2019).

Gallic acid (GA) serves not merely as a nutritional and healthcare aid in mitigating the effects and widespread occurrence of chronic liver diseases, but also enhances the prognosis of acute liver injury. GA, a phenolic acid, exhibits its antioxidant capacity primarily through the scavenging of free radicals and chelation of metals, a function attributed to its high-density phenolic hydroxyl groups. Additionally, GA is characterized by its high water solubility and a low risk of promoting oxidation. GA and procyanidin both belong to hydroxybenzoic acid polyphenolic compounds. Compared with procyanidin, GA has entered the clinical research stage through food supplements and is closer to practical application scenarios. Through the dissociation of phenolic hydroxyl groups, it directly neutralizes ROS, forms stable phenox free radicals, and its resonance structure can disperse electrons and avoid further oxidative damage (Tatipamula and Kukavica, 2021). In liver diseases, GA as an antioxidant, has high bioavailability, low toxicity, and the dual anti-injury effects of antioxidant and anti-apoptosis, especially outperforming quercetin, chlorogenic acid, and resveratrol in NASH models and acute liver injury. In terms of structure, GA contains 3 phenolic hydroxyl groups, which can efficiently scavenge free radicals. Although the phenolic hydroxyl groups of quercetin, belonging to the flavonol class, are widely distributed, molecular spatial hindrance may hinder its reaction efficiency (Tuli et al., 2022). Compared with other phenolic compounds, GA also known as 3,4,5-trihydroxybenzoic acid, is a natural compound widely present in various fruits and plants such as grapes, tea, and pomegranates. It is easily accessible and can be extracted using mature methods like acid hydrolysis, alkali hydrolysis, fermentation, and enzymatic processes, which are relatively low-cost and suitable for large-scale application (Simón et al., 2020). In the future, further research on GA can be conducted, developing its nano-delivery system (such as liposome encapsulation) to enhance liver targeting and improve the clinical transformation potential.

GA is a natural phenolic compound, has shown long-term potential for development (Mansouri et al., 2013). In 1786, Carl Wilhelm Scheele, a chemist, discovered and extracted GA from the fructus schisandrae. GA was primarily sourced from natural gallic acid and sumac fruits (Fernandes and Salgado, 2016). In the treatment of various diseases, GA has a potential therapeutic effect. GA plays a multifaceted role in alleviating heart disease through various biological pathways. GA reduces myocardial hypertrophy induced by transforming growth factor beta 1 and dysfunction caused by transverse aortic constriction, while also decreasing deposition of type I collagen, thereby preventing cardiac fibrosis (Jin et al., 2018). GA acts to mitigate cardiac hypertrophy remodeling and heart failure resulting from transverse aortic coarctation, accomplishing this by alleviating inflammation, oxidative stress, and fibrosis within cardiomyocytes (Yan et al., 2019). Moreover, GA plays a crucial role in mitigating inorganic phosphate-induced vascular smooth muscle cell calcification by blocking the BMP2-Smad1/5/8 signaling pathway (Kee et al., 2014). As a treatment for cancer, GA has a variety of powerful anti-cancer effects. In non-small cell carcinoma, GA inhibits the activation of the epidermal growth factor receptor (EGFR) and prevents coactivator-associated arginine methyltransferase 1 (CARM1) from binding to proline, glutamate, and leucine-rich protein 1 (PELP1). This decreases the formation of the CARM1-PELP1 complex and inhibits its growth (Wang and Bao, 2020). In breast cancer, GA inhibits the phosphoinositol-3 kinase (PI3K)/Akt pathway and reduces the accumulation of β-catenin, thus alleviating cancer symptoms (Hong et al., 2023). In gastric cancer, GA inhibits the epithelial-mesenchymal (EMT) transition, blocking the further development of GPL through the Wnt/beta-catenin signaling pathway (Liao et al., 2023). With the study of GA, the chemical synthesis method of GA is further adopted and designed, so that GA can be produced in large quantities. Consequently, GA has been widely used in pharmaceuticals and industrial production due to its wide range of applications (Xiang et al., 2024). In medicine, GA is considered a promising drug due to its powerful anti-inflammatory and anti-oxidant properties, as evidenced by its use in various medical applications (Kahkeshani et al., 2019).

Until now, GA research has mainly been focused on inflammation (Bai et al., 2021), cancer (Verma et al., 2013), diabetes (Xu et al., 2021c), intestinal disease research (Yang et al., 2020). However, the protective effects of GA on liver diseases and the underlying mechanisms remain unclear. Given the extensive clinical evidence and expert recommendations for the use of GA and its derivatives in treating liver diseases, it is imperative to further investigate their pharmacological effects, mechanisms, and potential significance in the management of these conditions. In order To better understand the role of GA in various liver diseases, this article reviews the effects of GA in the treatment of different degrees of liver diseases, such as viral hepatitis and alcoholic liver disease, and the mechanism of action, including its anti-inflammatory, antioxidant, and immunomodulatory effects. This review have significantly advanced our understanding of GA and its derivatives, elucidating their role and mechanisms in liver diseases. This progress may pave the way for novel treatment strategies for liver conditions in the future. Moreover, little information is available on GA in clinical research, so we can undertake in-depth studies to develop a more reliable basis for GA’s future application in clinical applications.

## Methods

### Search strategy

This review employed the Preferred Reporting Items for Systematic Reviews and Meta-Analyses (PRISMA) guidelines to conduct a comprehensive search of relevant literature. Multiple keywords were used to search for literature related to gallic acid, liver diseases, liver toxicity, hepatotoxicity, liver injury, alcoholic liver disease, non-alcoholic fatty liver disease, liver cancer, hepatocellular carcinoma, drug-induced liver injury, liver fibrosis, pharmacology, and safety in PubMed, Web of Science, Google Scholar, and Scopus.

### Inclusion and exclusion criteria

**FIGURE 1:**
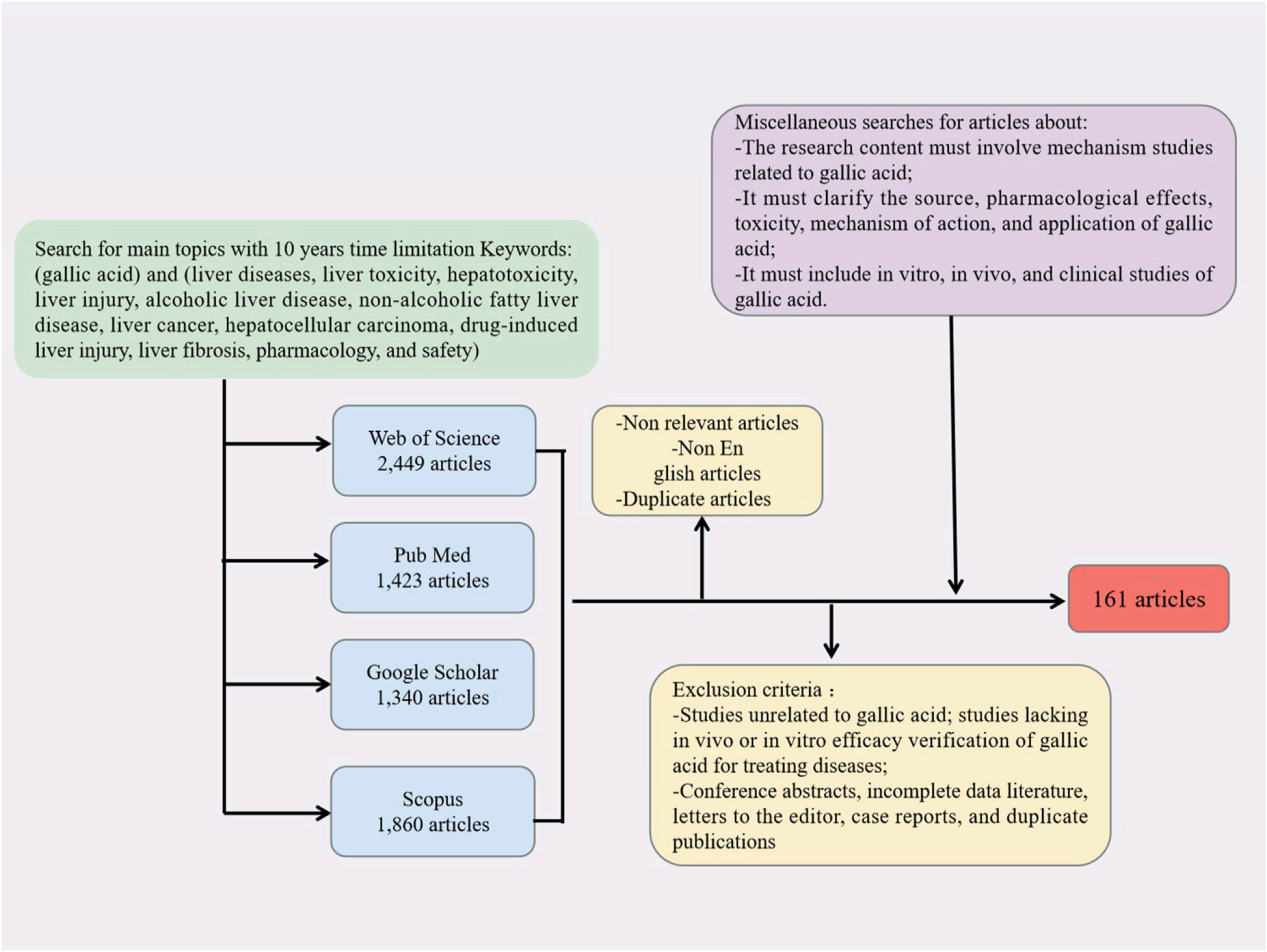
Flow chart depicting the search process, along with the criteria used for inclusion and exclusion.

The inclusion criteria for data screening were as follows: the research article must be published in English; the research content must involve mechanism studies related to gallic acid; it must clarify the source, pharmacological effects, toxicity, mechanism of action, and application of gallic acid; it must include *in vitro*, *in vivo*, and clinical studies of gallic acid. The exclusion criteria included: studies unrelated to gallic acid; studies lacking *in vivo* or *in vitro* efficacy verification of gallic acid for treating diseases; conference abstracts, incomplete data literature, letters to the editor, case reports, and duplicate publications. The search process flow chart is shown in Figure 1.

## Phytochemistry of GA

### The source of the gallic acid

GA was first isolated and identified by Scheele in 1786. It was initially obtained from the extract of oak gall and is a natural secondary metabolite mainly extracted from various fruits, plants and nuts (Wianowska et al., 2023). GA is commonly found in fruits and vegetables, such as grapes, bananas, mushrooms, and olives, and can be obtained from non-sugar gallates in our diet (Habibi et al., 2016; Tang et al., 2018; Wianowska and Olszowy-Tomczyk, 2023; Woldegiorgis et al., 2014; Yuan et al., 2017). It is also widely present in many medicinal plants, including kudzu root, schisandra berries, and Rhodiola (Türkan et al., 2020; Wang et al., 2019; Wei et al., 2020). Certain nuts, including cashews, walnuts, and hazelnuts, can also serve as sources for isolating and extracting GA (Daglia et al., 2014). Moreover, GA is present in tea leaves and grains (Qi et al., 2022), and previous studies have also reported its presence in fruit wines, such as wine (Sterneder et al., 2021; Xu et al., 2021b). In nature, GA occurs as a hydrolyzed tannin, also known as 3, 4, 5-trihydroxybenzoic acid, typically existing as a monohydrate with the molecular formula C_7_H_6_O_5_. Its relative molecular mass is 188.12 g per molecule. In most cases, GA is colorless or pale yellow (Keyvani-Ghamsari et al., 2023). With the in-depth study of GA, we are gradually exploring some of its derivatives. GA derivatives fall into two main categories, GA esters and catechin derivatives. It is worth noting that derivatives of GA have some of the same effects as GA, and even modified derivatives have improved effects over GA. For example, epigallocatechin-3-gallate (EGCG), derived from tea leaves, maintains human health. Its polyphenolic structure grants it strong antioxidant capacity. EGCG is hydrophilic rather than lipophilic. Therefore, EGCG can be esterified with long-chain saturated fatty acids and unsaturated fatty acids, and the structure can be modified to ultimately produce the lipated EGCG derivatives. The increased lipid solubility through the octanol-water partition coefficient can enhance the absorption of a drug by the body (Zhong and Shahidi, 2011). At present, GA derivatives have also been applied to the food industry and pharmaceutical industry, which also provides a better choice for the treatment of some diseases. GA has been reported to be obtainable through chemical or biosynthetic methods, resulting in an improved extraction yield of GA (Bai et al., 2021).

### The absorption and utilization of GA

GA occurs in varying concentrations across different foods, including blueberries, strawberries, grapes, mangoes, cashews, walnuts, tea, and wine. Upon ingestion, about 70% of GA is absorbed and subsequently metabolized, primarily into 4-O-methyl gallocatechin (4-OMeGA) (Daglia et al., 2014). This metabolite is widely distributed in the human body, particularly in the kidneys, lungs, and liver, and this extensive distribution may be related to the various potential biological activities and pharmacological effects of GA. Interestingly, despite its widespread distribution in various tissues, GA exhibits a relatively low content in brain tissue (Ma et al., 2016). Moreover, the detection of other GA metabolites in urine by researchers underscores the diverse nature of its metabolism (Pereira-Caro et al., 2017). In conclusion, a thorough understanding of the absorption and metabolic processes of GA is essential for uncovering its broader implications. These processes are influenced by various factors, including absorption pathways, the distribution of metabolites, as well as the regulatory effects of external and internal factors. Future researches are expected to uncover the potential health benefits and pharmacological mechanisms of GA.

In order to enable the GA to be better absorbed and utilized by the human body, When utilizing GA, we should primarily avoid concurrent consumption with copper-rich foods, as this combination can lead to rapid oxidation and degradation of the compound. Copper ions expedite the oxidation of GA, highlighting the significant impact of metal ions on the stability of plant phenols. The adjacent triphenolic group of GA can simultaneously form multiple hydrogen bonds and coordination bonds with metal ions, making it easier to be oxidized in the complex and generating stable dimer and polymeric products. Therefore, it should be avoided to be used together with high-copper foods such as animal liver, oysters, and shellfish. Iron-rich foods have a relatively weak effect on GA, but long-term and large amounts of consumption still require attention. The interactions of other foods (such as high-protein and alkaline foods) lack direct data and are not yet listed as major restrictions (Nkhili et al., 2014). These findings provide a theoretical basis for understanding the antioxidant mechanism of phenols in food and the influence of metal ions.

## Protective effects of GA in liver disease

Studies have shown that GA can provide a degree of protection against different stages of liver disease. This paper will discuss and elaborate in detail (Table 1).

**TABLE 1:** Information about the mechanism of action of GA on the liver disease.

Disease	Animal/cell model	Types	Routes	Dose	Effects and related mechanisms	References
NAFLD 1-9	Dust-induced NAFLD male Wistar rats	*in vivo*	po	100 mg/kg	↓TG, MDA, TNF-α, IL-6, HO1, NF-κβ, ALT, AST, ALP, cholesterol, miRs122, miRs34a ↑TAC	Fanaei et al. (2021)
	N-nitrosodiethylamine-induced NAFLD male Wistar rats	*in vivo*	po	100 mg/kg	↓ALT, AST, ALP.ROS, γ-GT	Chen et al. (2011)
	HFD and STZ-induced NAFLD male C57BL6/J mice	*in vivo*	po	150 mg/kg	↓ALT, AST, TG ↑insulin, 3-hydroxybutyrate, Xanthine, Lactate, PUFA, PUFA/UFA	Chao et al. (2020)
	HFD-induced NAFLD C57BL/6J mice HepG2 and SMMC-7721 cells	*in vivo* *in vitro*	ig	50,100,200 mg/kg 20 µM	↓AST, ALT, TG, TC, LDL-C ↑HDL-C	Zhang et al. (2023)
	Fructose-induced NAFLD male C57BL/6J mice HepG2 and Huh-7 human hepatoma cell lines	*in vivo* *in vitro*	ig	100,200 mg/kg 0, 25, 50, 100 μM	↓AST, ALT, TC, TG, LDL-C, MDA, ROS, COX-2, TNF-α, IL-6 ↑HDL-C, GSH-Px, T-SOD activity, Nrf2, HO-1	Lu et al. (2023)
	PA and OA-induced NAFLD in HepG2, Hepa 1–6, RAW 264	*in vitro*		50,100,200 µM	↓CD36, ACCα, SREBF-1, LXR-α, FATP-2, Bcl-2 mRNA expression	Tanaka et al. (2020)
	HFD-induced NAFLD male C57BL/6 mice	*in vivo*	ig	15,30 mg/kg/d	↓insulin, HOMA-IR, TC, TG, LDL-c.ALT, AST, IL-6, IL-1beta, IL-2, TNF-α ↑HDL-c, SOD, MDA	Lü et al. (2023)
	HFD-induced NAFLD male C57BL/6 mice	*in vivo*	ig	25,300 mg/kg/d	↓Hmgcr, Acc1, Abca1, Mttp, Cd36, Glu, TG, TC, ALT	Kant et al. (2020)
	HFD-induced NAFLD male C57BL/6 mice	*in vivo*	ig	50,100 mg/kg	↓Glucose, Triglyceride, Cholesterol, HDL, AST, insulin, HDL AST, ALT ↑PUFA/MUFA	Chao et al. (2014)
ALD 10-12	Ethanol-caused hepatocyte injury in LO2 cell	*in vitro*		15,30,60 μM	↓AST, ALT, LDH, RIP1, RIP3, HMGB1 ↑NRF2	Zhou et al. (2019)
	Ethanol-caused injury hepatocyte male C57BL/6J mice	*in vivo*	ig	100,200,400 mg/kg	↓AST, ALT, γ-GT, Serumiron, TS, liver iron, MDA, TC, TG ↑UIBC, SOD, GSH	Wu et al. (2019)
	Ethanol-caused hepatocyte injury female albino rats of Sprague–Dawley	*in vivo*	ig	50,100 200 mg/kg	↓ALD, LDH, AST ↑PON, Arylesterase	Kartkaya et al. (2013)
Liver fibrosis 13-18	CCl4-induced liver fibrosis male BALB/c mice	*in vivo*	ig	5.15 mg/kg	↓HA, cIV, MDA, ALT, AST, γ-GT, MMP-2 ↑TIMP-1	Wang et al. (2014)
	TAA-induced Liver fibrosis male Sprague-Dawley rats HSC-T6 cell, hepatocytes	*in vivo* *in vitro*	ig	50 mg/kg 0,12.5,25,50,100,200, 300 μg/mL	↓ALT, AST, MDA, collagen, deposition, PDGF-BB ↑caspase-3, GSH, TGF-β1, α-SMA, PCNA	El-Lakkany et al. (2019)
	DMN-induced liver fibrosis male Sprague Dawley rats	*in vivo*	ig	25, 50, 100 mg/kg	↓AST, ALT.ALP, TB, MDA, EGF, α-SMA, PDGFR, TIMP-1, TIMP-2, TGF-β1, Hydroxyproline, α-SMA ↑CAT, T-SOD, GSH	Chen et al. (2018)
	TAA-induced liver fibrosis male albino Wistar rats	*in vivo*	po	20 mg/kg	↓ALT, AST, ALP, MDA, TGF-β1, miR-21 ↑miR-30, miR-200, SOD	Hussein et al. (2020)
	HFD-induced liver fibrosismale wistar rats TNF-α-induced insulin-resistant FL83B mouse hepatocytes	*in vivo* *in vitro*	po	10,30 mg/kg 6.25 μg/mL	↓phospholipids, TAG, cholesterol, LDL cholesterol, insulin, leptin, GSSG ↑glutathione, glutathione reductase, glutathione peroxidase, glutathione S-transferase	Huang et al. (2016)
	CCL_4_-induced liver fibrosis male Wistar albino rats	*in vivo*	ip	50,100 mg/kg	↓TG, TC, ALT, AST, GGT ↑p53, GSH	Perazzoli et al. (2017)
DILI 19-33	DIC-induced liver toxicity male Wistar rats	*in vivo*	po	50,100 mg/kg	↓protein carbonyl, AST, ALP, ALT, total bilirubin, MDA, serum IL-1β ↑GSH, GPx, SOD, CAT	Esmaeilzadeh et al. (2020)
	Dox-induced hepatotoxicity Male Wistar rats	*in vivo*	po	60,120 mg/kg	↓ALT, ALP, Totao bilirubin, MDA, TT, CAT, H_2_O_2_ ↑GSH, GPx, GST, NPT, NO	Omobowale et al. (2018)
	INH-induced hepatotoxicity RFP-induced hepatotoxicity Male Wistar rats	*in vivo*	ig	50,100,150 mg/kg	↓ROS, MDA, LDH, MDA, TNF-α, IL-1β	Sanjay et al. (2021)
	TAA induced hepatotoxicity male albino rats	*in vivo*	po	100 mg/kg	↓ALT, AST, ALP, TNF-α, IL-6, NF-kβ, FAS, CASP-3, Glucose, GGT, DB, TB, TC, TG, NH_3,_ MDA, FAS ↑GSH, 5-HT, NE, DA, Albumin	Mohamed and Hafez (2023)
	t-BHP-induced hepatotoxicity in L02 cell	*in vitro*		20, 40 μM	↓DCF, Fluorescent Intensity, GSSG, ROS, LDH ↑GCLC, HO-1	Feng et al. (2018)
	Cisplatin-Induced hepatotoxicity male Wistar albino rats	*in vivo*	ig	8 mg/kg	↓8-OHdG, WBC, AST, ALT, Creatinine, Albumin ↑GSH, CAT, SOD, RBC, HGB, HCT, PLT	Doğan, Meydan, and Kömüroğlu, (2022)
	Fluoxetine-induced liver damage male Wistar rats	*in vivo*	po	50, 100, 200 mg/kg	↓MDA, GOT, GPT, Protein carbonyl, TNF-α ↑CAT, Vit C, SOD, plasma FRAP	Karimi-Khouzani, Heidarian, and Amini, (2017)
	Paracetamol-induced hepatotoxicity male crossbreed Swiss albino mice	*in vivo*	ig	100 mg/kg	↓ALT, AST, ALP, Lipid peroxidation ↑Total reduced glutathione, Glutathione reductase Glutathione peroxidase, Glutathione-S-transferase Catalase, Superoxide dismutase	Rasool et al. (2010)
	SA-induced hepatotoxicity male Wistar albino rats	*in vivo*	ig	10,30 mg/kg/day	↓ALT, AST, ALP, BUN, Cr, MDA, NO, IL-1β ↑GSH, GPx, SOD, CAT	Gholamine et al. (2021)
	CCL4-induced hepatic damage male Wistar rats	*in vivo*	ig	50,100 mg/kg	↓IL-1B, IL-6, COX2, TNFα, ALT, AST, ALP, GGT ↑SOD, CAT	Ojeaburu and Oriakhi, (2021)
	CCL4-induced hepatic damage male Sprague Dawley rats	*in vivo*	ig	100 mg/kg	↓AST, ALT, TBARS, MDA, CYP2E1 ↑GRD, GPX, CAT, GSH/GSSG, SOD	Tung et al. (2009)
	CCL4-induced hepatic damage male albino Wistar rats	*in vivo*	po	50 mg/kg	↓ALT, AST, ALP, LDH, TBARS ↑SOD, CAT, GPx	Hsouna et al. (2016)
	AFB1-induced hepatorenal dysfunction male Adult Wistar rat	*in vivo*	po	20,40 mg/kg	↓TNF-α, IL-1, RONS, LPO, Caspase 3, NO, MPO ↑IL-10, SOD, GPx, GST, GSH	Owumi et al. (2020a)
	Mn-induced hepatotoxicity male Wistar rats	*in vivo*	ig	15,30 mg/kg	↓AST, ALT, ALP, LDH, GGT, Serum creatinine, Serum urea ↑SOD, CAT, GST, GPx, GSH	Owumi et al. (2020b)
	iron-overload-induced liver injury male C57BL/6J mice	*in vivo*	ig	200 mg/kg	↓liver iron concentration, TS, UIBC, TIBC, TC, TG, AST ALT, γ-GT ↑MDA, SOD, GSH	Wu et al. (2023)
HCC 34-39	N-nitrosodiethylamine- induced HCC male Wistar rats	*in vivo*	po	50 mg/kg	↓AFP, GPC-3, STAT3 ↑SOCS3	Aglan et al. (2017)
	Male BAB/c nude mice HepG2, Bel-7402, LO2 cell	*in vivo* *in vitro*	ip ig	80 mg/kg 80 120 μM	↓MALAT1, Bcl-2, Bcl-xl, VEGF, Oct3/4, survivin, CCND1, Vimentin, MMP9 ↑E-cadherin	Shi et al. (2021)
	HepG2 cell	*in vitro*		550 µM	↓IL-8 ↑IL-10, IL-12	Lima et al. (2016)
	HepG2 SMMC - 7721 cell	*in vitro*		6.25,12.5, 25 μg/mL	↓Bcl-2 ↑caspase-3, caspase-9, ROS, Bax	Sun et al. (2016)
	DEN-induced HCC male Wistar rats	*in vivo*	ig	20 mg/kg	↓AFP, AST, ALT, γ-GT, globulin, L-MDA, bilirubin, NF-κB p65, MDA, Bcl-2, GGT ↑CAT, GSH, CASPASE-3	AboZaid et al. (2024)
	Hep3B, HepJ5, Mahlavu cell	*in vitro*		10,20,40 μg/mL	↓Bcl-2 ↑ROS, superoxide, fATG5/12, Beclin-1, c-PARP, caspase3, Bax, Bad	Huang et al. (2021)
Liver Cirrhosis 40-41	CCL4-Induced Hepatic Cirrhosis male C57 mice	*in vivo*	ip	100 mg/kg/day	↓NF-κB, p38 MAPK, Procollagenα1(I) ↑TGF-β1, TIMP1, p65	Figueiredo et al. (2017)
	BDL-Induced Hepatic Cirrhosis male Wistar rats	*in vivo*	ig	20,30 mg/kg	↓GGT, ALP, AST, ALT, IL-6, TNF-α, MDA, casepase-3, triglyceride, cholesterol, total billirubin ↑SOD, CAT	Jafaripour et al. (2023)

### Effect of GA in non-alconolic fatty liver disease (NAFLD)

NAFLD is a series of liver diseases, which is closely related to type 2 diabetes mellitus, obesity, and hyperlipidemia. It can range from simple steatosis to inflammation and fibrosis, and eventually lead to irreversible liver cirrhosis (Tanaka et al., 2020). Studies have shown that GA can improve antioxidant capacity and protect against oxidative stress in the liver (Perazzoli et al., 2017). Dust has been shown to induce NAFLD in rats by causing oxidative stress and triggering oxidative stress in inflammatory pathways in an experimental study by Hafseh Fanaei et al. In comparison to rats exposed to dust, the administration of GA was found to enhance the liver’s antioxidant capacity and reduce malondialdehyde (MDA) levels, indicating a protective effect against oxidative stress. Moreover, GA has been shown to mitigate liver damage in rats exposed to dust, significantly reducing the levels of aspartate aminotransferase (AST), alanine aminotransferase (ALT), and alkaline phosphatase (ALP), and altering the serum triglyceride (TG) levels, similar to the protective effects observed with PM2.5 exposure. Decreased the levels of anti-oxidative and pro-inflammatory cytokines (HO-1, Nrf2, IL-6, NF-κB and TNF-α) (Fanaei et al., 2021). Results indicate that GA has protective effects on the liver, preventing non-alcoholic fatty liver disease induced by a high-fat diet (HFD). Based on an evaluation of HFD-induced measures of liver fat, GA significantly reduced dyslipidemia, hepatic steatosis, and oxidative stress *in vivo*. Further research found that GA can reverse the decline of peroxisome proliferator-activated receptor alpha (PPAR-α) and liver X receptor-alpha (LXR-α) and the increase of sterol regulatory element-binding protein-1c (SREBP-1c) mRNA caused by HFD by anti-oxidative stress and improving steatopathy, thus alleviating NAFLD (Chen et al., 2011). A model of HFD and STZ-induced hyperglycemia and non-alconolic fatty liver disease showed that GA alleviated lipid accumulation and upregulation of -oxidation and ketogenesis, thereby alleviating hyperglycemia (Chao et al., 2020).

GA has the potential to alleviate NAFLD via the AMPK pathway, subsequently hindering the progression to hepatitis and cirrhosis, thereby underscoring AMPK as a crucial therapeutic target. When GA is administered, AMPK is phosphorylated, and acetyl-CoA carboxylase 1 (ACC1) and ACC2 are both inactivated by AMPK, therefore inhibiting the conversion of acetyl-CoA to malonyl-CoA, thus blocking fat synthesis (J et al., 2023). In a study conducted by Yuzhen Lu et al., GA showed anti-steatosis ability in a fructose-induced NAFLD mouse model with a hepatic qualitative phenotype. GA downregulated liver and serum triglyceride levels and reversed the abnormal levels of liver enzymes (ALT and AST) and lipid indices (TC, LDL-c and HDL-c) in the presence of GA. GA can enhance AMPK phosphorylation in a dose-dependent manner, inhibit the transcriptional activity of SREBP-1 and its downstream fatty acid synthase (FASN) and ACC to downregulate lipogenesis, thereby inhibiting hepatic lipogenesis and accumulation and reducing hepatic steatosis in fructose-induced NAFLD mice (Lu et al., 2023). Moreover, in another study showed that GA inhibited the expression of SREBP-1c and LXR α, key transcription factors that regulate fatty acid and triglyceride synthesis, while also downregulating the fatty acid transporter CD36, gene expression of fatty acid transport protein (FATP 2) and ACCα attenuated FFA uptake and lipogenesis, thereby improving hepatic steatosis. The results also showed that GA decreased the protein expression of precursor and mature SREBP-1c, while increasing the phosphorylation of AMPK and subsequently inhibiting SREBP-1c expression. Furthermore, the inactivation of SREBP-1c and LXR α had a significant impact on the downregulation of CD36, FATP 2, and ACC α expression induced by GA. In addition to the effect on lipogenesis, GA can also decrease the Bax/Bcl-2 mRNA expression ratio, thereby mitigating apoptosis and retarding NAFLD progression (Tanaka et al., 2020). The compound 1,2,3, 6-4-o gallic acyl-β-D-glucopyranoside, isolated from the peel of water caltrop, has been evaluated for its potential impact on NAFLD, similar to how other natural compounds such as those found in pomegranate peels have been studied for their effects on NAFLD. Studies have demonstrated that GA not only significantly reduces the levels of AST, ALT, TC, TG, and LDL-c induced by a high-fat diet (HFD), but also restores the levels of HDL-c, thereby playing a protective role for the liver. This is in line with the findings that high-density lipoprotein cholesterol (HDL-c) plays a crucial role in cardiovascular health, as evidenced by its protective effects against ASCVD. Research has demonstrated that GA modulates the AMPK/SREBP/ACC and IRs-1/Akt signaling pathways, which are pivotal in cellular metabolism, and it also suppresses liver inflammation by targeting the IKK/i-κB/NF-κB signaling pathway, a key mediator of inflammatory responses (Lü et al., 2023). Moreover, the natural polyphenol 1,2,3,4,6 penta-o-galloyl--D-glucose (PGG), which is derived from gallic acid, may also protect liver function in some studies. In the case of NAFLD, PGG has demonstrated the potential to alleviate the condition by reversing insulin resistance and reducing hyperlipidemia, as evidenced by clinical trials showing significant reductions in liver fat content with treatments such as Efinopegdutide and lanifibranor. PGG treatment has been shown to counteract the effects of a high-fat diet (HFD) by normalizing the expression of genes linked to lipid accumulation in the liver, thus offering a protective effect on liver health (Kant et al., 2020).

Previous studies on the mechanism of action of GA on NAFLD were based on pharmacological methods. This study delves deeper into the beneficial impacts of GA administration on hepatic steatosis, employing animal models and nuclear magnetic resonance metabolomics for a more thorough and meticulous analysis. The results of liver histopathology and liver serum biochemistry examined in NAFLD mice show that GA administration prevents hepatic adipose deformation, insulin resistance, and hypercholesterolemia induced by HFD. Furthermore, GA has been shown to reverse metabolic disorders associated with NAFLD and exhibits a beneficial preventative impact on metabolic disorders overall. Elevated levels of ALT and AST in the serum of HFD mice serve as a crucial indicator of liver damage. In addition, the levels of TG, cholesterol and fatty acids in the liver of HFD mice were also significantly increased, and the ratio of monounsaturated fat (MUFA) to polyunsaturated fat (PUFA) was decreased. Following GA administration, liver TG and cholesterol levels were reduced, d fatty acid levels decreased, the ratio of PUFA to MUFA increased, and the levels of ALT and AST decreased significantly. In conclusion, GA can improve liver steatosis induced by HFD in NAFLD mice (Chao et al., 2014).

In summary, we have delved into the mechanisms of GA in NAFLD. Recent studies suggest that traditional Chinese medicine, including compounds like GA, holds significant therapeutic potential for NAFLD by modulating lipid metabolism disorders, reducing lipid accumulation, and decreasing the level of cellular apoptosis. The potential of GA as a pharmacological treatment for NAFLD and NASH is highlighted by recent research, which underscores the need for further investigation into its efficacy and mechanisms of action. Currently, there is limited research on GA in NAFLD, and its underlying mechanisms warrant further investigation.

### Effect of GA in alcoholic liver disease (ALD)

ALD is a chronic liver disease caused by long-term alcohol abuse, including different stages such as fatty liver, alcoholic hepatitis and cirrhosis. The pathogenesis primarily involves factors such as cellular damage, inflammation, and oxidative stress, which may ultimately lead to liver failure and liver cancer. ALD is one of the most common liver diseases worldwide, causing acute alcoholic hepatitis in severe cases with concomitant high mortality (Louvet and Mathurin, 2015; Seitz et al., 2018; Singal et al., 2018).

In the study by Zhou et al., GA inhibited ethanol-induced hepatocyte necrotizing apoptosis through Nrf2 induction, demonstrating its beneficial effects on alcoholic liver disease (Zhou et al., 2019). Furthermore, in the mouse model of alcoholic liver disease, the soy lecithin-gallocatechin complex has also been proven to be effective in reducing oxidative stress, lipid peroxidation and liver fibrosis. According to another study, GA increases liver PON1 (paraoxonase1) and arylesterase activity via the intracellular signaling cascade PPAR-PKA-cAMP. Levels of liver paraoxonase, arylesterase, ALT, AST, and LDH can serve as indicators to evaluate the degree of liver damage. After the administration of GA, these indexes decreased significantly, indicating that GA can improve liver damage caused by alcohol-induced oxidative stress, so GA also plays an important protective role in ALD (Kartkaya et al., 2013).

**FIGURE 2:**
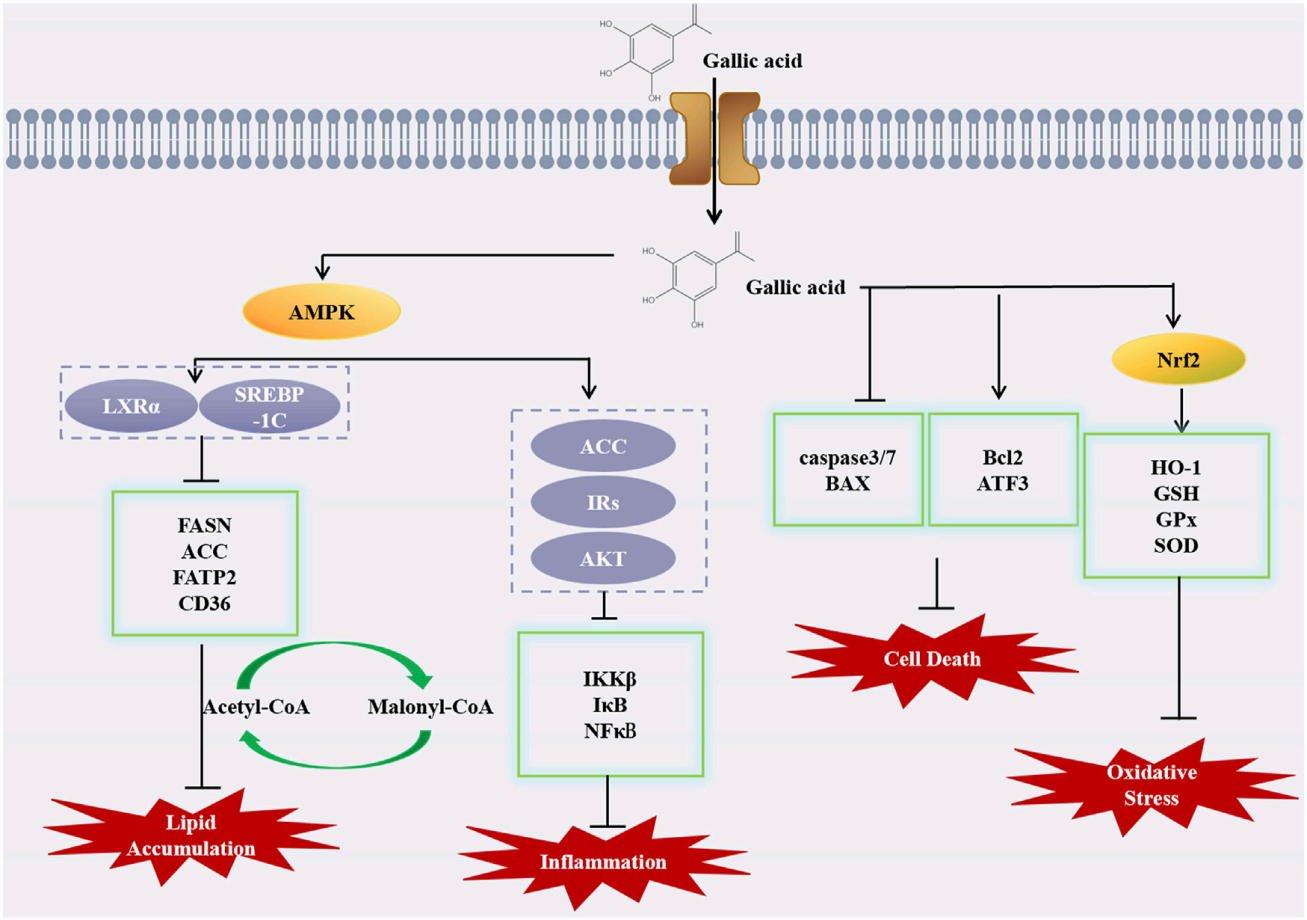
Diagram of anti-fatty liver (including NAFLA and ALD) mechanism of GA.

In conclusion, GA and its derivatives may alleviate alcohol-induced liver injury through different mechanisms, and GA has significant protective effects on alcohol-induced ALD (Figure 2). The above studies can further explore the specific dosage of GA and provide a new direction for the treatment of alcoholic liver disease.

### Effect of GA in liver fibrosis

Liver fibrosis, a stage in the progression towards cirrhosis, is a result of chronic inflammation and tissue repair responses following repeated liver injury. It can be caused by a variety of factors including chronic hepatitis, autoimmune disorders, alcohol-related liver disease, non-alcoholic liver diseases, and other conditions. The global prevalence of advanced liver fibrosis and cirrhosis is significant, with a rising trend and notable geographical variations. For instance, China has one of the highest incidences and mortality rates of liver cancer, which is closely linked to liver fibrosis. It plays a pivotal role in contributing to global morbidity and mortality rates. Those suffering from chronic liver conditions are prone to developing liver fibrosis, thereby elevating their risk of cirrhosis and, potentially, hepatocellular carcinoma (Lai and Afdhal, 2019). So far, the role of GA in liver fibrosis has been investigated.

When administered to the treatment group of rats, GA demonstrated a dose-dependent reduction in fibrotic area, inflammatory infiltration, fat degeneration, as well as levels of hyaluronic acid (HA), type IV collagen (cIV), ALT, AST, and gamma-glutamyl transferase (γ-GT). Simultaneously, it decreased the expression of matrix metalloproteinases-2 (MMP-2) and tissue inhibitor of matrix metalloproteinases-1 (TIMP-1). It appears that GA inhibits carbon tetrachloride-induced liver fibrosis by deactivating hepatic stellate cells (HSC), thereby preventing disease progression (Wang et al., 2014). It is noteworthy that in another study, GA not only inhibited the activation of HSC but also suppressed the expression of proliferating cell nuclear antigen (PCNA), thereby impeding the proliferation of damaged liver cells (El-Lakkany et al., 2019). Furthermore, it decreased the expression of α-SMA and induced apoptosis in HSCs, which contributed to the mitigation of fibrosis progression *in vivo*. In another article, GA was shown to alter TGF-/Smad signaling pathway, thereby modifying dimethylnitrosamine-induced liver fibrosis in rats (Chen et al., 2018). Simultaneous administration of GA showed a significant improvement in liver fibrosis induced by thioacetamide (TAA) in rats. By inhibiting the pro-fibrotic miR-21 and upregulating the expressions of miR-30 and miR-200, GA can effectively prevent the fibrotic process. Its main mechanism of action lies in inhibiting the TGF-β1/Smad3 signaling pathway, thereby reducing the production of ROS, inhibiting the activation of hepatic stellate cells, reducing collagen synthesis, and delaying the progression of fibrosis. GA can reduce liver oxidative stress by improving HFD-induced obesity in rats, which can delay the occurrence of liver fibrosis (Hussein et al., 2020). In HFD-induced obese rats, GA can also reduce levels of phospholipids, TAG, cholesterol, LDL cholesterol, insulin, and leptin. In addition, GA can also reduce liver oxidative stress and GSSG levels in HFD-induced rats, while increasing the levels of glutathione, glutathione reductase, glutathione peroxidase and glutathione S-transferase. These findings suggest that GA has a marked effect on reducing the incidence of obesity, oxidative stress, and liver fibrosis in rats induced by HFD (Huang et al., 2016). In one study, it was found that GA can reduce the occurrence of tetrachloride (CCL4) induced liver fibrosis by increasing the expression of p53 gene, because the increase of p53 gene expression will accelerate the apoptosis process of damaged liver cells. At the same time, p53 also has antioxidant effects, and its increased expression will increase the content of liver glutathione, thereby reducing liver oxidation and restoring cell function, and playing a role in improving liver peroxidation. After treatment with GA, the abnormal liver function caused by carbon tetrachloride can be significantly improved, and the production of free radicals can be reduced, so as to alleviate and improve liver fibrosis (Perazzoli et al., 2017). GA is a hydrophilic compound. When GA is encapsulated within a capsule delivery system and formulated into reverse micelles (RMs), which are then encapsulated in lipid nanocapsules (LNCs), it enhances the efficiency of hydrophilic groups and minimizes drug leakage. At present, nanotechnology is highly applicable to the treatment of liver fibrosis, especially when targeting liver stellate cells. The reverse micellar-loaded lipid nanocapsules (RMLNC), formulated through the Box-Behnken design and preparation method, enable direct delivery of GA to activated hepatic stellate cells, effectively suppressing their proliferation, activation, and migration. GA-RMLNC is an extremely effective method for treating liver fibers, and more *in vivo* studies can demonstrate the safety and effectiveness of the delivery system (Radwan et al., 2020).

**FIGURE 3:**
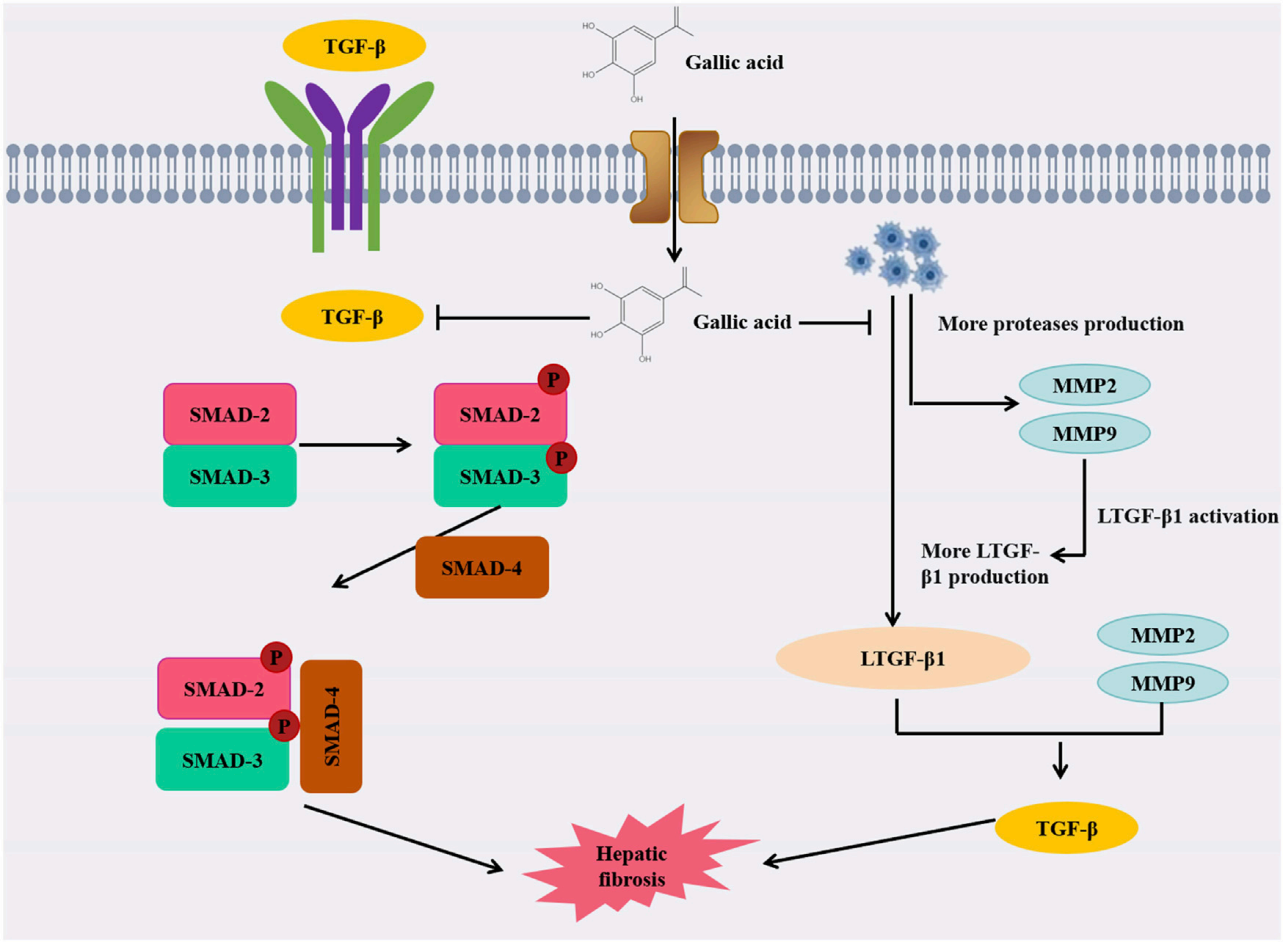
Diagram of anti-liver fibrosis mechanism of GA.

Collectively, these findings suggest that GA could be effective in treating liver fibrosis via a variety of mechanisms, alleviating the severity of the condition (Figure 3). Looking ahead, these findings pave the way for future research in the field of liver fibrosis treatment. Further investigations could delve into optimizing dosage regimens for GA administration, exploring potential synergistic effects with other therapeutic agents, and examining long-term effects on liver function and fibrotic progression.

### Effect of GA on drug-induced liver injury (DILI)

The phenotype of DILI is intricate, encompassing nearly all presently recognized forms of liver injury, ranging from mild, asymptomatic elevations in liver biochemical markers to jaundice, acute liver failure, and in the most severe cases, fatality (Li et al., 2022b). The predominant cause behind the majority of drug development failures or the withdrawal of drugs from the consumer market is often attributed to a combination of factors, including insufficient clinical efficacy, toxicity issues, and poor drug-like properties (Ding and Yang, 2019). Currently, clinical treatment of DILI continues to face substantial challenges, GA has shown promising inhibitory effects on DILI, as demonstrated by several studies.

Diclofenac (DIC) is a non-steroidal anti-inflammatory medicine commonly used for treating rheumatoid arthritis pain. Metabolites derived from cytochrome P450-dependent DIC oxidation (2,5-quinone imines and 4,5-hydroxy-diclofenac) can enhance reactive oxygen species (ROS) production by inducing redox imbalance and mitochondrial dysfunction. This increases the risk of liver injury. In rats induced by DIC, liver levels of GSH, GPx, SOD, and CAT were markedly reduced, while serum protein carbonyl, AST, ALP, ALT, total bilirubin, MDA, serum IL-1β, and liver IL-1β gene expression levels were significantly elevated. However, these parameters were reversed in rats treated with GA (Esmaeilzadeh et al., 2020). In addition, GA pretreatment ameliorated doxorubicin (Dox) -induced hepatotoxicity and oxidative stress. The contents of glutathione and non-protein thiol (NPT), glutathione peroxidase (GSH-Px) and glutathione S-transferase significantly increased. The serum levels of ALT, MDA, hydrogen peroxide production, superoxide dismutase, catalase, alkaline phosphatase and total bilirubin significantly decreased (Omobowale et al., 2018). When used inappropriately, isoniazid and rifampin, which are commonly prescribed for tuberculosis, can cause serious liver injury and even death. In response to anti-tuberculosis drug treatment, GA may reduce the elevation of liver function enzymes, liver necrosis and inflammation. GA prevented isoniazid and rifampicin cytotoxicity by increasing the activation of Nrf2 and induction of its downstream targets and inhibiting the NF-κB/TLR-4 axis upregulated by anti-tuberculosis drugs (Sanjay et al., 2021). It is believed that GA can significantly ameliorate liver injury caused by thioacetamide (TAA), particularly by regulating the NF-κB signaling pathway. GA can reduce the levels of inflammatory markers, including TNF-α, IL-6. By supplementing GSH, GA can decrease MDA levels in the liver. It can also reduce the levels of FAS, caspase-3, AST, ALT, GGT and ALP to alleviate liver inflammation and oxidative stress. Its purpose is to mitigate liver damage resulting from TAA-induced necrosis, fibrosis, and congestion among other conditions (Mohamed and Hafez, 2023). GA, through the activation of the ERK signaling pathway, enhances Nrf2-mediated antioxidant responses, safeguarding liver cells and mitigating hepatotoxicity induced by t-BHP (Feng et al., 2018). The application of GA can also reduce the production of ROS and increase the level of GSH in cells, thereby reducing the t-BHP-induced hepatotoxicity. The most common side effects of cisplatin include kidney and liver toxicity, along with other side effects that can endanger people’s lives. It is not only a drug, but also used in the treatment of malignant tumors that gives serious side effects. When cisplatin induces oxidative stress, GA reduces MDA and 8-OHdG levels while increasing GSH levels and thereby reducing liver toxicity (Doğan et al., 2022). In clinical practice, fluoxetine is used as an antidepressant because it is a selective 5-HT reuptake inhibitor. Studies have shown that long-term use in depressed patients can cause serious liver damage. GA has been shown to mitigate fluoxetine-induced liver injury. Research findings indicate that GA’s hepatoprotective effect is linked to its ability to decrease ROS production, alleviate oxidative stress, and mitigate inflammation-related factors like tumor necrosis factor-α, thereby diminishing inflammation (Karimi-Khouzani et al., 2017). Paracetamol is a commonly used antipyretic drug. When paracetamol is taken excessively, it can be harmful to your liver. Excessive use of paracetamol depletes glutathione reserves, resulting in the accumulation of N-acetyl-p-benzoquinone imine (NAPQI), which disrupts mitochondrial function and can ultimately cause severe liver damage. Furthermore, paracetamol significantly elevates lipid peroxidation levels, thereby intensifying oxidative stress in the liver. After administration of GA peroxidase levels (SOD, CAT, GSH-Px, GSR, GST) are reversed, thereby reducing oxidative stress in the liver. GA can inhibit the release of serum tumor necrosis factor-α (TNF-α) induced by acetaminophen, which helps to slow down the progression of liver diseases (Rasool et al., 2010). GA can inhibit the liver and kidney toxicity induced by sodium nitrite (SA) in rats by improving the levels and histological changes of ALT, AST, ALP, Cr and BUN in serum. GA increases the level of GSH and the activities of SOD, CAT and GPx, and decrease the levels of MDA, NO and IL-1b in liver and kidney tissues. SA damages the liver primarily through the antioxidant effect of GA, improves intracellular antioxidant capacity, and finally plays a protective role in kidneys and liver (Gholamine et al., 2021). For CCL4 induced liver injury, GA not only downregulates pro-inflammatory cytokines, thereby inhibiting NF-κB signaling pathway, but also stimulates Nrf2-mediated antioxidant enzymes to reduce liver injury induced by CCl4 poisoning in rats (Ojeaburu and Oriakhi, 2021). In another study, GA significantly alleviated mild liver toxicity induced by CCL4 in rats, as evidenced by a substantial reduction in plasma TBARS levels, inhibition of cytochrome P450 activation, and attenuation of oxidative stress in the liver. Following the administration of GA, the activity of antioxidant enzymes such as superoxide dismutase (SOD), glutathione reductase (GRD), glutathione peroxidase (GPX), and catalase (CAT) in liver tissue is observed to increase. This elevation in enzyme activity enhances the liver’s antioxidant defense mechanisms, thereby contributing to the reduction of liver damage (Tung et al., 2009). GA extracted from spinipless plum fruit, the main component of this fruit, can reduce the increase of TBARS level after CCl4 administration, restore the activity of antioxidant enzymes (SOD, CAT and GPx), reduce the liver cell damage caused by oxidative stress, prevent the liver toxicity induced by CCl4, and play a role in liver protection (Hsouna et al., 2016). Aflatoxin is a potent carcinogen, and GA has a protective effect against oxidative and inflammatory stress induced by aflatoxin B1 (AFB 1) in rats and liver. GA has the ability to decrease the synthesis of pro-inflammatory biomarkers in the liver and lower IL-10 levels. It also enhances the activity of antioxidant enzymes and boosts intracellular glutathione levels, thereby reducing oxidative stress and promoting liver health (Owumi et al., 2020b).

In addition to drugs, excessive amounts of heavy metals can also induce severe liver damage. For instance, exposure to manganese can decrease the levels of GSH and the activity of antioxidant enzymes like CAT, GPx, SOD, and GST in rat models. It is evident from the lower levels of these enzymes that the liver does not perform its antioxidant function efficiently. GA plays a protective role in liver oxidative stress induced by manganese exposure and lipid peroxidation through antioxidant stress. The levels of AST and ALT in liver serum also decreased significantly after GA administration, indicating that GA has a certain protective effect on manganese-mediated liver injury (Owumi et al., 2020a). Excessive iron will produce a large number of reactive oxygen species, exacerbate the occurrence of oxidative stress, and even cause liver cell damage or death, resulting in abnormal liver function. Due to GA’s poor fat solubility, it struggles to penetrate cell membranes, thereby limiting its bioavailability ability is poor. In order to improve bioavailability, GA and soybean lecithin complex (SL-GAC) can be synthesized to improve bioavailability and increase pharmacological activity. Iron overload can lead to increased levels of MDA, TC, and TG, which in turn cause oxidative stress in the liver. This highlights that SL-GAC can protect liver tissues from damage caused by iron overload (Wu et al., 2023).

**FIGURE 4:**
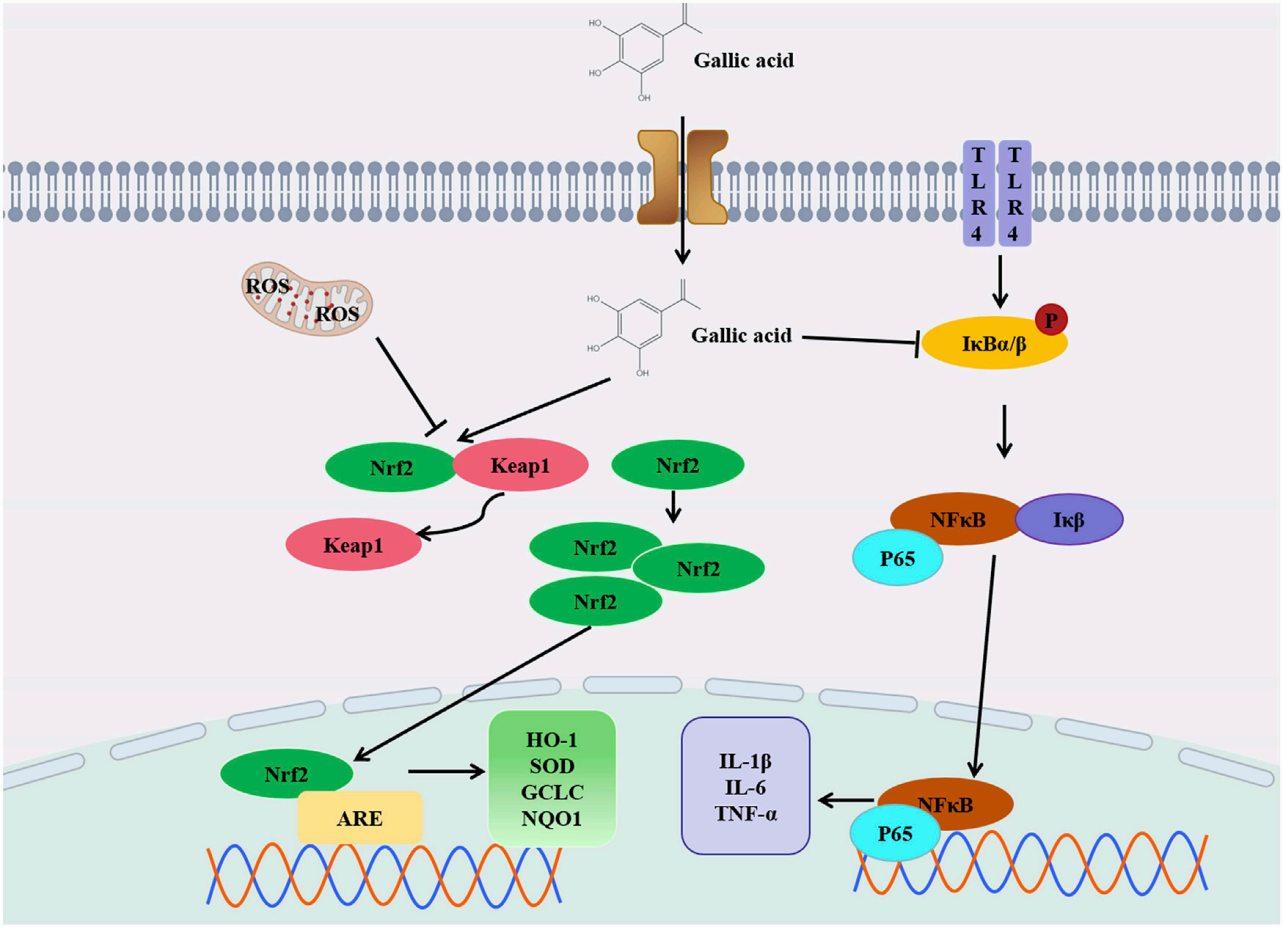
Diagram of anti- DILI mechanism of GA.

In summary, the research findings unequivocally indicate that GA holds significant potential in the prevention and treatment of drug-induced liver injury. Through in-depth exploration of its antioxidant, anti-inflammatory, and cytoprotective properties, we have discovered that GA exerts a notable effect in alleviating drug-induced liver damage (Figure 4). This discovery provides crucial clues for the development of novel hepatoprotective drugs or therapeutic strategies, underscoring the promising prospects of GA in the realm of liver health. Nevertheless, further research is required to delve into its detailed mechanisms and assess the feasibility of clinical applications, ensuring its maximum efficacy in the treatment of drug-induced liver injury.

### Effect of GA in hepatocellular carcinoma (HCC)

Liver cancer, an aggressive form of cancer affecting the liver, frequently occurs alongside chronic liver diseases and cirrhosis. It ranks as the fifth most prevalent cancer among men and the seventh among women globally. According to the latest data, liver cancer is the third leading cause of cancer-related deaths worldwide. Typically, hepatocellular carcinoma (HCC) is detected at a late stage, which significantly worsens the prognosis. Over 90% of liver cancer cases are HCC, for which chemotherapy and immunotherapy are preferred treatments, albeit posing therapeutic challenges. Therefore, exploring new therapeutic avenues, such as natural. The application of nanotechnology in the treatment of hepatocellular carcinoma (HCC) is essential for providing more effective therapeutic options, promising to improve patient outcomes and reduce side effects. Recent research has shown that GA holds promise in the treatment of HCC, offering new avenues for therapeutic intervention (Anwanwan et al., 2020; Liu et al., 2015).

**FIGURE 5:**
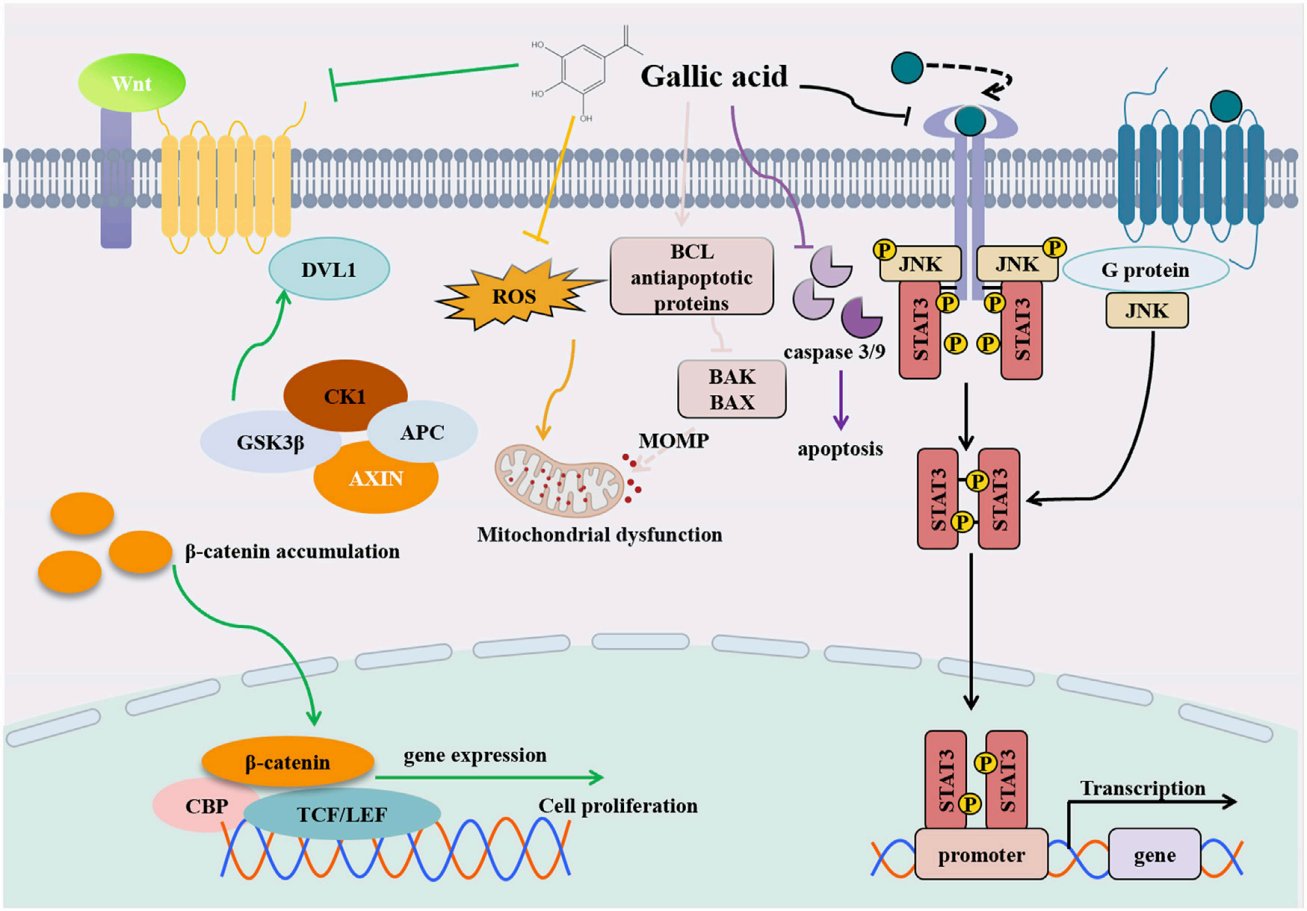
Diagram of anti- HCC mechanism of GA.

A study by Hee Young Kwon et al. demonstrated that GA treatment led to a reduction in the levels of AFP, GPC-3, and STAT3 in liver serum, while increasing the level of suppressors of cytokine signaling 3 (SOCS3). This suggests that GA may have a protective effect against hepatocellular carcinoma, as GPC-3 is a specific biomarker for liver cancer and its decreased expression could indicate a reduction in cancerous activity. This protective effect may be mediated through the STAT3 signaling pathway (Aglan et al., 2017) (Figure 5). Chuan-jian Shi and colleagues observed that GA significantly decreased the viability of HepG2 and Bel-7402 cells in a concentration-dependent manner. Simultaneously, GA induced cell cycle arrest and apoptosis in HCC cells, effectively suppressing their migration (Shi et al., 2021). Furthermore, GA attenuated Wnt/β-catenin signaling activity and downregulated metastasis associated lung adenocarcinoma transcript 1 (MALAT1) expression in HCC cell (Lima et al., 2016) (Figure 5). Interestingly, the study also revealed that the overexpression of MALAT1 partially reversed the inhibitory effects of GA on proliferation and migration, successfully alleviating the inhibition of Wnt/β-catenin signaling. In summary, these findings underscore the significant potential of GA in the treatment of HCC, particularly in its potential application to inhibit tumor development through its influence on the MALAT1-Wnt/β-catenin axis. During Sun’s experiments, SMMC-7721 cells and hepatocytes were exposed to varying concentrations of GA (0, 6.25, 12.5, and 25.0 μg/mL) to assess the compound’s impact. In SMMC-7721 cells, GA augmented caspase-3, caspase-9, and ROS production, reducing mitochondrial outer membrane permeabilization (MOMP) production, and eventually causing apoptosis. Remarkably, GA had no impact on HL-7702 hepatocyte apoptosis. Based on these findings, GA appears to have selective anticancer properties by causing apoptosis specifically in SMMC-7721 cells (Sun et al., 2016). Utilizing microwave-assisted extraction, active components such as GA were efficiently extracted from pomegranate peel. These components were then used as raw materials to synthesize GA-coated zinc oxide nanoparticles (Zn-GANPs), as detailed in the study. This zinc oxide nanoparticle is a possible anticancer nanoparticle that can deliver anticancer drugs targeted to tumor sites without toxicity to normal tissues. Diethylnitrosamine (DEN) is a carcinogen that can damage the liver and induce cancer. DEN mediates the occurrence of inflammation and the regulation of biological processes by increasing reactive oxygen species. It causes severe liver damage. The administration of zinc nanoparticles coated with gallic acid (Zn-GANPs) has been shown to significantly ameliorate the severity of liver oxidative stress, inflammation, and histopathological changes, as well as restore normal liver function. Zn-GANP was found to elevate malondialdehyde (MDA) levels while simultaneously reducing the expression of caspase-3, the activity of antioxidants such as glutathione (GSH) and catalase (CAT), the levels of inflammatory markers including alpha-fetoprotein (AFP) and nuclear factor kappa B p65 (NF-κB p65), and the concentrations of transaminases alanine aminotransferase (ALT) and aspartate aminotransferase (AST). Recent studies have shown that zinc nanoparticles coated with gallic acid (Zn-GANPs) exhibit a preventive and therapeutic effect on liver injury induced by diethylnitrosamine (DEN), as evidenced by the work of AboZaid et al. (2024). In addition to GA, its derivatives have also shown to be effective in treating cancer. In a study by Chien-Yu Huang et al., a derivative compound of ellagic acid called ellagic acid methyl ester (MG) was shown to increase cellular superoxide and oxidative stress levels. It can induce autophagy to inhibit cancer cell proliferation, which is associated with the activation of caspase-3 and the modulation of the levels of ligands such as Bcl-2, Bax, and Bad (Huang et al., 2021).

In summary, research suggests that GA and its derivatives hold promise for treating HCC. Through a multitude of mechanisms including the inhibition of cell growth, induction of cell cycle arrest and apoptosis, and interference with crucial signaling pathways such as the MALAT1-Wnt/β-catenin axis, GA exhibits substantial potential in the therapy of HCC. These findings provide promising clues for the development of new therapeutic strategies for HCC, thereby offering hope for improving the often grim prognosis of patients with HCC and reducing the occurrence of treatment side effects. Nevertheless, in order to apply these findings effectively in clinical practice for patients with HCC, further research and clinical trials are needed.

### Role of GA in liver cirrhosis

Liver cirrhosis is a prevalent condition among liver diseases, with both the incidence and mortality rates gradually increasing in recent years (Tsochatzis et al., 2014). The occurrence of hepatic cirrhosis is influenced by various factors such as excessive alcohol intake, obesity, type 2 diabetes, hepatitis B and C infections, which can further lead to liver fibrosis and scar formation, ultimately damaging the normal structure and function (Sepanlou et al., 2020). Studies have demonstrated that GA can reduce oxidative stress in the liver, thereby reducing the occurrence of liver fibrosis and further cirrhosis.

In the study of Sergio Souza Figueiredo et al., it was found that GA was able to inhibit ROS-induced oxidative stress, thereby alleviating the occurrence of liver fibrosis. Meanwhile, GA not only decreases the expression of α1 procollagen (I), TGF-β1, and TIMP1 genes but also inhibits signaling pathways mediated by TGFβ1, p65 NF-κB, and p38 MAPK. This dual mechanism contributes to decreasing the incidence of liver fibrosis and cirrhosis (Huang et al., 2023; Figueiredo et al. (2017)). In addition, another study found that GA has been shown to restore bile acid stasis, lipid peroxidation, inflammatory cytokines, and caspase-3 expression in liver tissues, which are adversely affected by bile duct ligation. GA mitigates oxidative stress and inflammation, alleviates hepatocyte necrosis, and counters liver fibrosis, thereby enhancing the condition of liver cirrhosis stemming from bile duct ligation. Furthermore, GA decreases liver enzyme levels, including GGT, ALP, AST, and ALT, while augmenting the expression of antioxidant genes, like SOD and CAT, thereby facilitating liver recovery. In this study, GA can also reduce the expression of IL-6 and TNF-α genes, thereby reducing the inflammation of liver tissue after biliary cirrhosis (Jafaripour et al., 2023).

Studies have clearly shown that GA exhibits a protective effect on liver cirrhosis, a condition regarded as an end-stage disease (Fabrellas et al., 2020). By mitigating oxidative stress from various inducing factors, GA can lessen hepatocyte necrosis and liver fibrosis. Current clinical approaches to treating GA in the context of liver cirrhosis remain unclear, with ongoing research needed to elucidate the specific mechanisms and efficacy of GA in this condition. Hence, GA presents a promising new avenue for the treatment of liver cirrhosis.

## Clinical applications and limitations of GA

GA can be used alone or in combination with certain drugs in clinical practice. When used in comb ination with certain drugs, it can produce a synergistic effect. GA can be used in combination with various antibiotics, demonstrating remarkable therapeutic efficacy in treating infections caused by drug-resistant bacteria. When combined with azithromycin (AZM), GA not only reduces the minimum inhibitory concentration (MIC) and enhances anti-biofilm activity but also promotes reactive oxygen species (ROS) generation, thereby significantly potentiating the antibacterial efficacy against methicillin-resistant *Staphylococcus aureus* (MRSA) (Khoshi et al., 2025). Combination therapy involving GA and carbenicillin (CAR) can lead to an elevation in the minimum inhibitory concentration (MIC) of CAR. This indicates that GA interferes with the antibacterial activity of carbenicillin and reduces its inhibitory effect on purple *bacillus* CV026. When GA is combined with tetracycline (TET), GA may reduce the bioavailability of tetracycline by binding to it; or it may change the physiological state of bacteria, causing a change in the sensitivity of bacteria to tetracycline, thereby affecting the antibacterial effect and biofilm formation, and reducing its inhibitory effect on purple *bacillus* CV026 (Dusane, D. H et al., 2015). GA can also significantly enhance the antibacterial activity of ciprofloxacin and erythromycin against multiple human and avian-derived enteric *vibrio* strains (including drug-resistant strains). There may be a synergistic effect between GA and donepezil, especially in improving the oxidative stress induced by aluminum chloride (AlCl3) neurotoxicity (Obafemi et al., 2021). These studies provide new ideas for the clinical application of GA.

GA boasts wide application, efficient oral absorption, and rapid elimination post-administration. In animal experiments, GA was found to have almost no toxicity. GA demonstrates efficacy not only in liver diseases but also in kidney, heart, and brain disorders, among others (Bai et al., 2021). Given its robust antioxidant properties, GA serves as a valuable antioxidant in both the food and pharmaceutical industries. However, GA’s disadvantage lies in its limited stability. To enhance the stability of GA, optimizing its structure is a viable approach. GA exhibits a low bioavailability, a challenge that can be effectively addressed through the application of nanotechnology. Techniques such as emulsions and nanoparticles have been shown to significantly enhance the solubility and stability of drugs, thereby improving their bioavailability (Keyvani-Ghamsari et al., 2023; Vaishali et al., 2022). There are two main derivatives of GA, one as an ester and the other as a catechin. Compared to GA, derivatives of GA may exhibit enhanced drug effects due to their improved lipophilicity and increased cell membrane permeability. Therefore, we should continue to explore GA and its derivatives to establish a stronger foundation for future drug development and research.

## Conclusion and outlook

This review highlights the protective effects of GA against various liver diseases, such as fibrosis, non-alcoholic fatty liver disease (NAFLD), alcoholic liver disease (ALD), drug-induced liver injury (DILI), hepatocellular carcinoma (HCC), and cirrhosis. Studies have shown that GA can significantly improve liver fibrosis in rats induced by carbon tetrachloride (CCl4), suggesting its potential in preventing and treating liver diseases. GA alleviates these conditions via multiple mechanisms, such as activating Nrf2, inhibiting NF-κB/MAPK pathways, reducing oxidative stress, and suppressing inflammation, underscoring its therapeutic potential. Currently, research is ongoing into GA and its derivatives, yet a deeper understanding of their clinical application and disease-specific mechanisms is still needed. Although animal models show no GA toxicity, clinical safety assessments are pending. Continued research will strengthen their utility for liver disease therapy.

## Glossary

ACC1: Acetyl-CoA carboxylase 1
ALD: Alcoholic liver disease
ALP: Alkaline phosphatase
ALT: Alanine aminotransferase
AMPK: AMP-activated protein kinase
AST: Aspartate aminotransferase
CARM1: Coactivator-associated arginine methyltransferase 1
DIC: Diclofenac
DILI: Drug-Induced Liver Injury
EGCG: epigallocatechin-3-gallate
EGFR: Epidermal growth factor receptor
EMT: Epithelial-mesenchymal
FATP 2: fatty acid transport protein 2
FFA: Frontal fibrosing alopecia
GA: Gallic acid
γ-GT: Gamma-glutamyl transferase
HA: Hyaluronic acid
HCC: Hepatocellular carcinoma
HFD: High-fat diet
IL-1: Interleukin 1
IL-6: Interleukin 6
LNCs: Lipid nanocapsules
LXR-α: Liver X receptor-alpha
MDA: Malondialdehyde
MMP-2: Matrix metalloproteinases-2
NAFLD: Non-alcoholic fatty liver disease
NF-κB: Nuclear factor kappa B
Nrf2: Nuclear factor erythroid 2-related factor
4-OMeGA: 4-O-methyl gallic acid
PELP1: Proline, glutamate, and leucine-rich protein 1
PGG: Penta-o-galloyl--D-glucose
PI3K: Phosphoinositol-3 kinase
PON1: Paraoxonase1
PPAR-α: Peroxisome proliferator-activated receptor alpha
RMLNC: Reverse micellar-loaded lipid nanocapsules
SA: Sodium nitrite
SREBP-1c: Sterol regulatory element-binding protein-1c
TAA: Thioacetamide
TG: Triglyceride
TGF-β: Transforming growth factor-beta
TIMP-1: Tissue inhibitor of matrix metalloproteinases-1
TNFα: Tumor necrosis factor alpha
